# Sublethal and transgenerational effects of sulfoxaflor on the demography and feeding behaviour of the mirid bug *Apolygus lucorum*

**DOI:** 10.1371/journal.pone.0232812

**Published:** 2020-05-14

**Authors:** Zengbin Lu, Song Dong, Chao Li, Lili Li, Yi Yu, Shuyan Yin, Xingyuan Men

**Affiliations:** 1 Maize Research Institute, Shandong Academy of Agricultural Sciences/National Engineering Laboratory of Wheat and Maize/Key Laboratory of Biology and Genetic Improvement of Maize in Northern Yellow-Huai River Plain, Ministry of Agriculture and Rural Affairs, Jinan, China; 2 Institute of Plant Protection, Shandong Academy of Agricultural Sciences, Jinan, China; 3 College of Plant Protection, Shandong Agricultural University, Tai’an, China; Institut Sophia Agrobiotech, FRANCE

## Abstract

Sulfoxaflor, the first commercially available sulfoximine insecticide, has been used for the control of sap-feeding insect pests such as plant bugs and aphids on a variety of crops. However, its sublethal effects on the mirid bug *Apolygus lucorum*, one of the key insect pests of Bt cotton and fruit trees in China, have not been fully examined. Here, we evaluated the demography and feeding behaviour of *A*. *lucorum* exposed to sulfoxaflor. The leaf-dipping bioassay showed that the LC_10_ and LC_30_ of sulfoxaflor against 3^rd^-instar nymphs of this insect were 1.23 and 8.37 mg L^-1^, respectively. The LC_10_ significantly extended the nymphal duration and decreased the oviposition period by 5.29 days and female fecundity by 56.99% in the parent generation (F0). The longer duration of egg, 5^th^-instar nymphs, preadult, and male adult longevity were observed in the F1 generation (F1) at LC_10_. At the LC_30_, the duration of egg and 1^st^-instar nymph, female adult longevity, and oviposition period of the F1 were significantly shorter, while the nymphal duration in the F0 and duration of 5^th^-instar nymphs, preadult survival rate, and male adult longevity in the F1 significantly increased. The net reproductive rate (*R*_0_), intrinsic rate of increase (*r*), and finite rate of increase (*λ*) in the F1 were not significantly affected by these two concentrations, whereas the mean generation time (*T*) was lower at the LC_30_. Additionally, the probe counts and cells mixture feeding time were markedly lengthened by the LC_10_ and LC_30_, respectively, when *A*. *lucorum* nymphs exposed to sulfoxaflor fed on Bt cotton plants without insecticides. These results clearly indicate that sulfoxaflor causes sublethal effects on *A*. *lucorum* and the transgenerational effects depend on the tested concentrations.

## Introduction

Many new insecticides have been developed and commercially produced to improve environmental safety and human health and control insect pests more effectively [1,2]. Sulfoxaflor, the first sulfoximine insecticide, was discovered by Dow AgroSciences and became commercially available in 2012 [1,3]. It acts as an agonist of insect nicotinic acetylcholine receptors (nAChRs) [4,5] and performs well against many sap-feeding insect pests, such as aphids, whiteflies, and plant bugs, and even against several insect species that are already resistant to neonicotinoids and other insecticides class [1,3,6]. However, similar to other insecticides, in addition to the direct mortality induced by sulfoxaflor, there is a need to determine its sublethal effects on insect pests.

Sublethal effects are defined as effects on survival individuals exposed to an insecticide [7, 8]. Numerous studies have shown that sublethal effects of insecticides affected the survival [9–11], developmental duration [12–18], and fecundity [19–22] of insects. For example, the 0.2 mg L^-1^ of imidacloprid prolonged the juvenile development and shortened reproductive period, adult longevity and fecundity of *Aphis glycines* (Hemiptera: Aphididae) [13]. Additionally, sublethal effects of insecticides also impaired the feeding behaviour of exposed insects, such as *Halyomorpha halys* (Heteroptera: Pentatomidae) [23], multiple aphid species [14, 19, 24–26], and *Diaphorina citri* (Hemipetera: Psyllidae) [27]. *H*. *halys* adults exposed to sulfoxaflor have lower feeding sites in soybean seeds (*Glycine max* (L.) Merr.) [23]. Therefore, sublethal effects of insecticides such as sulfoxaflor on arthropods should be completely examined to provide useful information on their integrated management.

The age-stage, two-sex life table and EPGs are two useful tools to assess the population-level performance and feeding behaviour of insects [26–29]. This kind of life table approach considers both sexes and stage differentiation and can estimate population parameters more accurately than the traditional female age-specific life tables [29,30]. Chen et al. [15] showed that treatment with the LC_25_ of sulfoxaflor significantly delayed the mean generation time (*T*), but reduced the intrinsic rate of increase (*r*), finite rate of increase (*λ*), net reproductive rate (*R*_0_), and gross reproduction rate (*GRR*) of the F1 generation of *Aphis gossypii* (Hemiptera: Aphididae). EPGs were recently used to monitor feeding behavioural changes in insects after exposure to insecticides [25–27,31]. Koo et al. [31] found that sublethal concentrations of imidacloprid inhibited the ingestion of phloem sap by *A*. *gossypii*. Thus, these two methodologies could be adopted to provide comprehensive insights into the sublethal effects of sulfoxaflor on insects.

The polyphagous mirid bug, *Apolygus lucorum* (Hemiptera: Miridae), has become an economically important pest of cotton and many other crops with the expansion of Bt cotton planting area in China [32–34]. They cause significant yield loss and low quality in crops by feeding on the terminal meristems, squares, and fruits, resulting in stunted plants and the abscission of bolls and fruits [16,35]. Management of *A*. *lucorum* mainly relied on chemical insecticides [36,37]. The 50% water-dispersible granules (WDG) of sulfoxaflor have been registered for emergency use in cotton fields in China to control mirid bug populations (www.chinapesticide.org.cn), with control effects of 85.6% even at 14 d after their spraying [37]. However, sulfoxaflor has exhibited sublethal effects on aphids [15,38], planthoppers [12], stink bugs [23], plant bugs [11], and psyllids [39], therefore it is necessary to assess its sublethal effects on *A*. *lucorum*. In the present study, we used the age-stage, two-sex life table and EPGs to investigate the sublethal effects of sulfoxaflor on the demography and feeding behaviour of the parent generation (F0 generation) of *A*. *lucorum* and its offspring (F1 generation). The aim was to fully understand the population fitness and feeding behaviour of this insect exposed to sulfoxaflor stress.

## Materials and methods

### Insects and plants

The overwintering eggs of *A*. *lucorum*, collected from a winter jujube orchard (37.79° N, 118.02° E) in Binzhou city, Shandong, China, in December 2016, were used to establish the laboratory colony and then maintained on green bean, *Phaseolus vulgaris* (Fabales: Fabaceae) in transparent glass jars (10 cm diameter × 15 cm height). These jars were kept in a climate-controlled chamber with a temperature of 25 ± 1°C, relative humidity (RH) of 65 ± 5%, and a photoperiod of L16: D8. The green beans were replaced every 7–10 days. Third-instar nymphs were used in the lethal toxicity test.

Bt cotton plants (*Gossypium* spp. var Lumianyan 36 (Malvales: Malvaceae), developed by Cotton Research Center, Shandong Academy of Agricultural Sciences, Jinan, China) were planted in plastic pots with soil and then placed in the above described chamber. Plants were watered as needed. Plant seedlings with 2–3 leaves were used in the feeding behaviour experiments.

### Insecticides

Closer^®^ (50% WDG, Dow AgroSciences, Shanghai, China), the commercial formulation of sulfoxaflor, was used in the following experiments.

### Lethal toxicity of sulfoxaflor to *Apolygus lucorum*

The leaf-dipping method was used to assess the lethal toxicity of sulfoxaflor to 3^rd^-instar nymphs of *A*. *lucorum*. Briefly, the formulation of sulfoxaflor was diluted with distilled water into five different concentrations: 5, 10, 25, 40, and 70 mg L^-1^. Distilled water was used as the control. Fresh green beans were washed, dried, and cut into 2-cm-long sections. These sections were then dipped into each concentration of sulfoxaflor or the control for 20 min and dried for 2 h in the laboratory. After that, they were placed into the transparent plastic box (6 cm diameter × 7 cm height) with two sections in each box. Fifteen 3^rd^-instar nymphs were added into each box and the box was then covered with plastic lids to prevent the insects from escaping. Each treatment was repeated three times. All boxes were placed in a climate-controlled chamber at standard environmental conditions (25 ± 1 °C, 65 ± 5% RH, L16:D8). Mortality was examined after 48 h and nymphs were considered dead if they did not move after being pushed with a soft brush.

### Sublethal effects of sulfoxaflor on the F0 generation of *Apolygus lucorum*

There were 155, 158, and 126 third-instar nymphs randomly selected from *A*. *lucorum* population reared in laboratory and exposed to each solution of the control, LC_10_ (1.23 mg L^-1^) and LC_30_ (8.37 mg L^-1^). After 48 h, the surviving nymphs were individually placed into the new smaller transparent plastic box (1.5 cm diameter × 2 cm height) with one insecticide-free green bean. The nymphal survival and development were observed and recorded daily. When the adults come out, the male and female from the same treatment was paired and each pair was transferred into a smaller transparent plastic box with one green bean section. Green beans were substituted with new ones daily. And the replaced beans were checked to count the number of eggs using a stereo microscope. When the male adults died, they were replaced with new ones from the same treatment group. The experiment would end until all adults were dead. All experiments were conducted under the conditions as the above described.

### Sublethal effects of sulfoxaflor on the F1 generation of *Apolygus lucorum*

To study the F1 generation, there were 117, 130, and 117 eggs from the F0 generation selected for the control, LC_10_, and LC_30_, respectively. Egg hatching was recorded daily and newly born nymphs were individually transferred into smaller transparent plastic boxes with one insecticide-free bean. The nymphal survival and development were observed and recorded every day. When adults emerged, the male and female from the same treatment were paired and the pair was transferred into a smaller transparent plastic box with one bean section. Green beans were substituted daily with new ones. And the replaced beans were checked to count the number of eggs using a stereo microscope. When the male adults died, they were replaced with new ones from the same treatment group. The experiment would end until all adults were dead. All experiments were conducted under the conditions as the above described.

### Sublethal effects of sulfoxaflor on the feeding behaviour of *Apolygus lucorum*

The probing and feeding behaviour of *A*. *lucorum* was recorded using a Giga-4 DC EPG amplifier (Wageningen, Netherlands) and a Faraday cage. Third-instar nymphs were first treated with the control, LC_10_ and LC_30_ prepared as above for 48 h, and subsequently, the surviving nymphs were transferred and starvation for 5 h. After that, these nymphs were fixed in a vacuum device and connected individually to gold wire (20 μm diameter × 2 cm length). One end of the gold wire was dipped in the water-soluble silver glue and pulled out many times until a small ball was formed. This ball was attached to the notum of each nymph under a stereomicroscope. The nymph was linked to the input probe of the EPGs. The plant electrode was inserted into the soil in the pots. The EPG signal of each nymph was monitored continuously for 6 h in Bt cotton plants using ANA 34 software (Wageningen, Netherlands). There were at least 20 nymphs tested on different plants for each treatment. Electrical signals were analysed and processed according to the previous references [40, 41].

### Data analysis

The LC_10_, LC_30_, and LC_50_ values were obtained with a Probit regression analysis. The development duration, adult longevity, and fecundity of the F0 generation of *A*. *lucorum* were analysed using Kruskal-Wallis test, since these data can not fulfil the assumption of normal distribution of residuals and homogeneity of error variances. Chi-squared test (*χ*^2^) was applied to compare the survival rate from 3^rd^-instar nymphs to adults of the F0 generation. The data on the feeding behaviour were analysed using one-way analysis of variance (ANOVA) followed by Tukey’s multiple comparisons. All statistical analyses were performed using SPSS 19.0 software (IBM, New York, USA).

The raw data in the F1 generation were analysed using the computer program TWOSEX-MSChart [42] based on the age-stage, two-sex life table theory [29, 30]. The age-stage-specific survival rate (*s*_*xj*_, the probability that a newborn will survive to age *x* and stage *j*), age-specific survival rate (*l*_*x*_, the probability of a newly laid egg surviving to age *x*), age-stage-specific fecundity (*f*_*xj*_, the mean number of eggs laid by a female at age *x* and stage *j*), and age-specific fecundity (*m*_x_, the mean fecundity of individuals at age *x*) were obtained from this computer program. The net reproductive rate (*R*_0_), intrinsic rate of increase (*r*), finite rate of increase (*λ*), and mean generation time (*T*) were calculated according to Chi and Liu [29] and Chi [30].

The standard errors of all the life history traits including survival, development duration, adult longevity, fecundity, and population parameters including *R*_0_, *r*, *λ*, and *T* in the F1 generation were calculated using the bootstrap technique with 100,000 resampling [43,44]. A paired bootstrap test was used to compare the means among treatments based on the confidence intervals at the 5% significance level [45]. All figures were created using SigmaPlot 14.0 software (Systat Software, Inc., CA, USA).

## Results

### Lethal toxicity of sulfoxaflor to *Apolygus lucorum*

The probit regression model derived from the concentration-morality results at 48 h was *Y* = 0.909*X*-1.363 (*χ*^*2*^ = 0.084, *P* = 0.994). The LC_10_, LC_30_, and LC_50_ of sulfoxaflor were estimated as 1.23 [95% confidence intervals: 0.02–3.91 mg L^-1^], 8.37 [1.66–15.06], and 31.57 mg L^-1^ [18.12–80.84], respectively.

### Sublethal effects of sulfoxaflor on the F0 generation of *Apolygus lucorum*

The development duration from 3^rd^-instar nymphs to adult emergence was significantly longer at both LC_10_ and LC_30_ compared with the control. In contrast, the LC_10_ significantly decreased the oviposition period by 5.29 days and female fecundity by 56.99% (Table 1). However, the nymphal survival rate from 3^rd^-instar to adult emergence, preoviposition period, and adult longevity did not significantly differ between sulfoxaflor treatment and the control group (Table 1).

**Table 1:** Sublethal effects of sulfoxaflor on biological parameters of the F0 generation of *Apolygus lucorum*.

Parameter	Control		LC_10_		LC_30_		Statistics	
	*n*	Mean ± SE	*n*	Mean ± SE	*n*	Mean ± SE	*H*	*P*
Development duration from 3^rd^ instar nymph to adult (days)	103	8.63±0.17b	79	9.76±0.24a	63	9.83±0.17a	22.11	*<*0.001
Survival of 3^rd^ instar nymph to adult (%)^a^		66.67a		50.00a		50.00a	*χ*^*2*^ = 2.95	0.229
Female adult longevity (days)	40	21.47±1.83a	32	16.56±1.62a	29	18.97±1.85a	4.45	0.108
Male adult longevity (days)	63	17.59±1.59a	47	14.77±1.69a	34	15.94±1.69a	1.44	0.487
Preoviposition period (days)	31	9.97±0.49a	17	12.53±1.06a	20	10.55±0.75a	4.25	0.120
Oviposition period (days)	31	14.00±1.36a	17	8.71±1.62b	20	11.05±1.61ab	6.08	0.048
Fecundity (eggs/female)	40	36.25±6.00a	32	15.59±3.78b	29	29.66±5.97ab	6.79	0.033

The same lowercase letters within the same row indicate that the treatments are not significantly different from each other based on Kruskal-Wallis test followed by post hoc multiple comparisons at *P ≤* 0.05.

^a^ Chi-square test

### Sublethal effects of sulfoxaflor on the F1 generation of *Apolygus lucorum*

**Fig 1:**
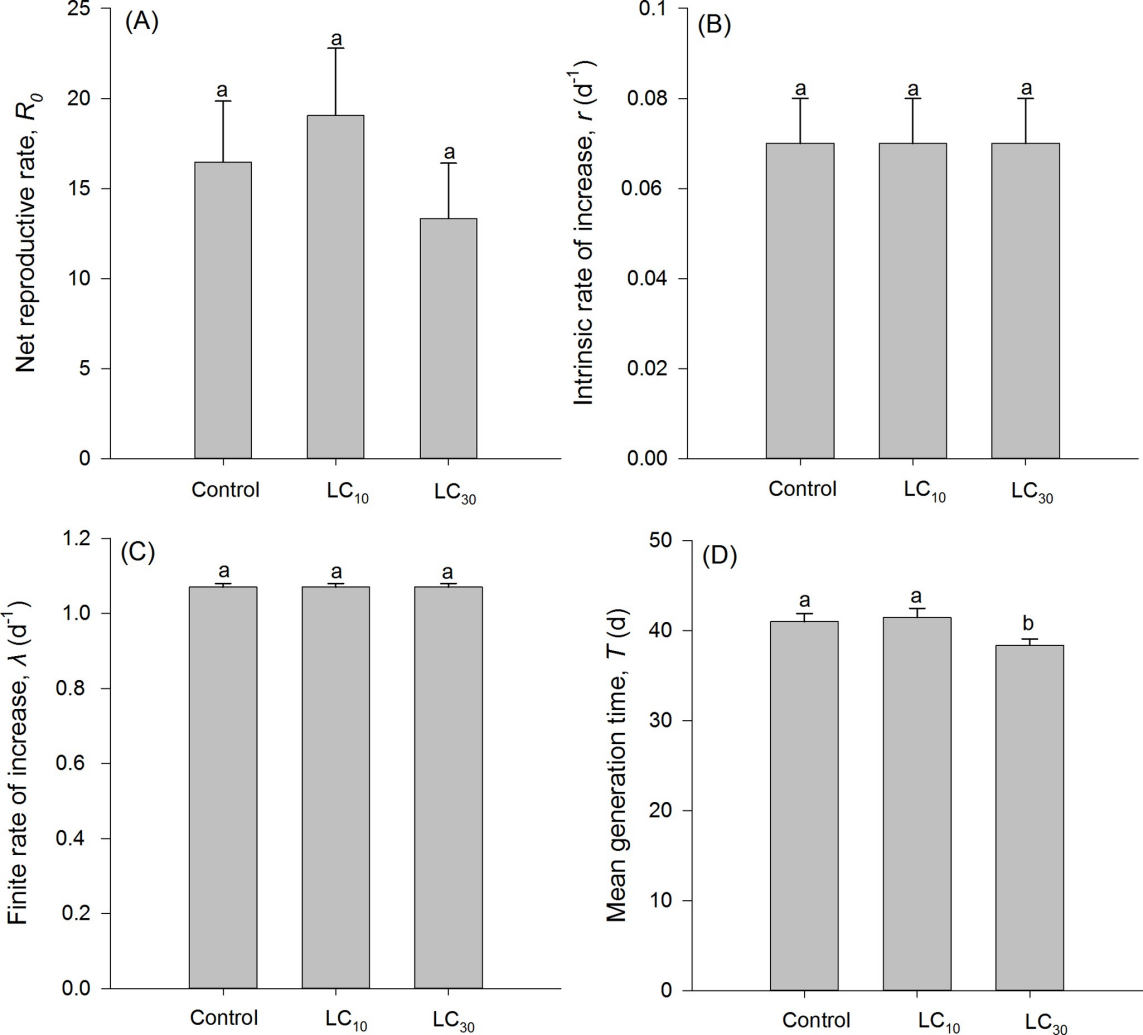
Sublethal effects of sulfoxaflor on the net reproductive rate (A), intrinsic rate of increase (B), finite rate of increase (C), and mean generation time (D) of the F1 generation of *Apolygus lucorum*. Different lowercase letters in the each parameter represent significant difference between treatments using a paired bootstrap test at the 5% significance level.

The biological parameters of the F1 generation of *A*. *lucorum* are presented in Table 2. Egg duration significantly increased by 0.25 days at LC_10_, but decreased by 0.86 days at LC_30_. Both of them dramatically extended the duration of 5^th^-instar nymphs and male adult longevity. Additionally, the preadult duration increased by 0.8 days at LC_10_ and the preadult survival rate was higher at LC_30_. By contrast, the duration of 1^st^-instar nymphs, female adult longevity, oviposition period, and mean generation time (*T*) were significantly reduced at LC_30_ (Fig 1). However, these concentrations did not significantly affect total nymphal development duration, adult preoviposition period (APOP), total preoviposition period (TPOP), fecundity, *R*_0_, *r*, and *λ* (Fig 1).

**Table 2:** Sublethal effects of sulfoxaflor on biological parameters of the F1 generation of *Apolygus lucorum*.

Parameter	Control		LC_10_		LC_30_	
	*n*	Mean ± SE	*n*	Mean ± SE	*n*	Mean ± SE
Egg duration (days)	92	7.79±0.08b	104	8.04±0.07a	94	6.93±0.06c
1^st^ instar nymph	76	2.55±0.09a	83	2.71±0.11a	85	2.33±0.06b
2^nd^ instar nymph	68	2.29±0.09a	81	2.51±0.10a	84	2.38±0.07a
3^rd^ instar nymph	65	2.23±0.09a	81	2.44±0.10a	81	2.30±0.06a
4^th^ instar nymph	61	2.69±0.10a	79	2.66±0.07a	78	2.85±0.07a
5^th^ instar nymph	50	4.92±0.11b	70	5.26±0.07a	72	5.33±0.10a
Total nymphal development duration (days)	50	14.78±0.21a	70	15.29±0.15a	72	15.17±0.13a
Preadult duration (days)	50	22.6±0.22b	70	23.4±0.18a	72	22.15±0.15b
Preadult survival rate		0.43±0.05b		0.54±0.04ab		0.62±0.05a
Adult preoviposition period (APOP) (days)	25	9.92±0.55a	30	9.37±0.63a	26	10.12±0.50a
Total preoviposition period (TPOP) (days)	25	32.52±0.71a	30	33.33±0.73a	26	32.12±0.61a
Oviposition period (days)	25	16.08±1.51a	30	16.73±1.35a	26	10.42±1.40b
Female adult longevity (days)	29	27.72±2.19a	33	27.52±1.60a	32	20.56±1.46b
Male adult longevity (days)	21	17.48±2.65b	37	23.97±1.46a	40	24.12±1.45a
Fecundity (eggs/female)	29	66.41±8.69ab	33	75.06±9.48a	32	48.84±8.56b

The standard errors (SEs) were estimated using the bootstrap technique with 100,000 resampling. Different lowercase letters in the same row represent significant difference between treatments using a paired bootstrap test at the 5% significance level.

**Fig 2:**
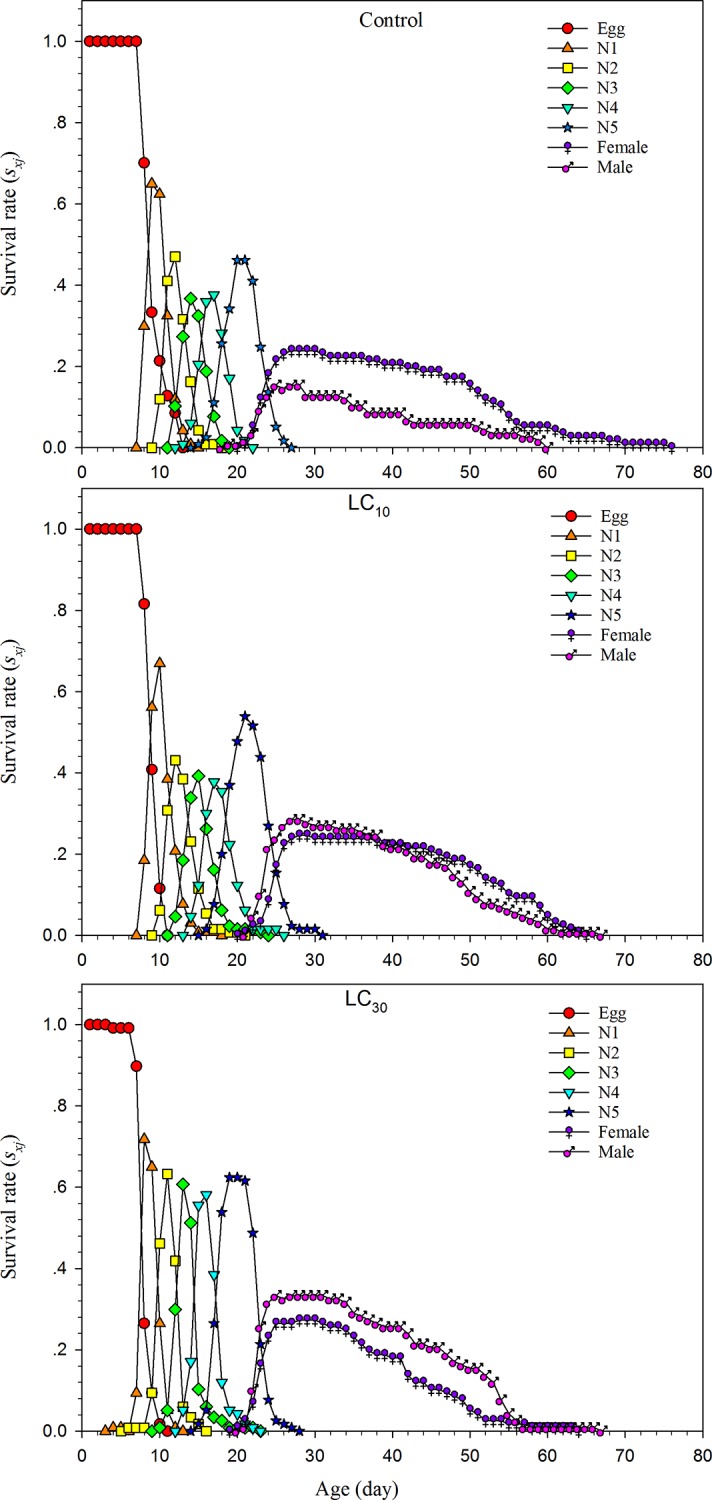
Sublethal effects of sulfoxaflor on the age stage-specific survival rate (*s*_*xj*_) of the F1 generation of *Apolygus lucorum*. N1-N5: 1^st^-instar nymph, 2^nd^-instar nymph, 3^rd^-instar nymph, 4^th^-instar nymph, and 5^th^-instar nymph, respectively.

The sublethal effects of sulfoxaflor on the age-stage-specific survival rate (*s*_*xj*_) of *A*. *lucorum* are presented in Fig 2. The overlaps between stages were observed in the control and sulfoxaflor owing to the different interindividual development rates. Moreover, in the control, both the survival rate and longevity of female adults increased compared with male adults, whereas the reversed effect was seen at LC_30_.

**Fig 3:**
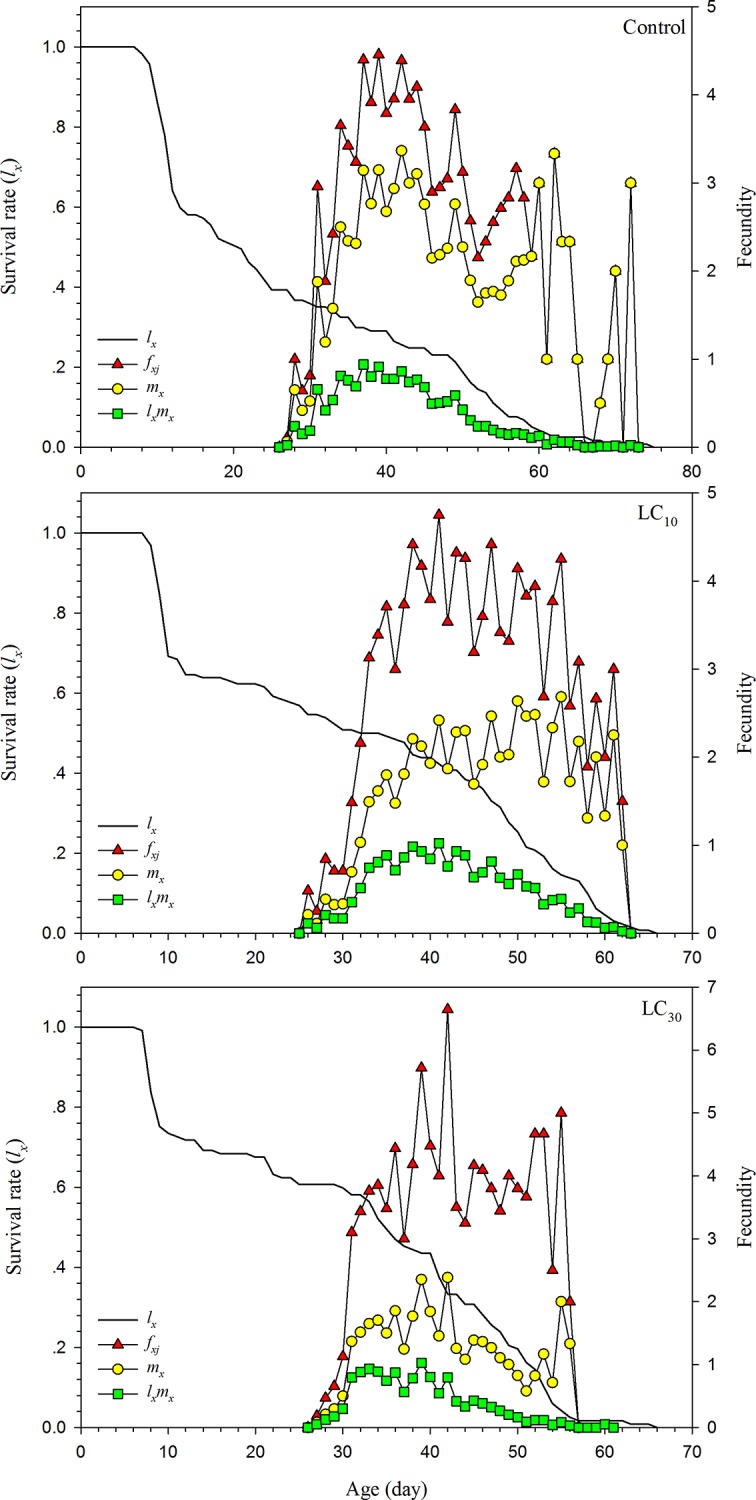
Sublethal effects of sulfoxaflor on the age-specific survival rate (*l*_*x*_), age-specific fecundity (*m*_*x*_), age-specific maternity (*l*_*x*_*m*_*x*_) and age-stage specific fecundity (*f*_*xj*_) of the F1 generation of *Apolygus lucorum*.

The sublethal effects of sulfoxaflor on the age-specific survival rate (*l*_*x*_), age-stage-specific fecundity (*f*_*xj*_), age-specific fecundity (*m*_*x*_), and age-specific maternity rate (*l*_*x*_*m*_*x*_) of *A*. *lucorum* are presented in Fig 3. The *l*_*x*_ dropped off as age increasing and the longest age of *A*. *lucorum* individuals for the control, LC_10_, and LC_30_ was 75, 67, and 65 days, respectively. The peaks of *f*_*xj*_ and *m*_x_ appeared at different times, being 4.46, 4.75, and 6.64 and 3.37, 2.68, and 2.38 eggs per female for the control, LC_10_, and LC_30_, respectively. The highest values of *l*_*x*_*m*_*x*_ were 0.94, 1.02, and 1.03 for the control, LC_10_, and LC_30_, respectively.

### Sublethal effects of sulfoxaflor on the feeding behaviour of *Apolygus lucorum*

**Fig 4:**
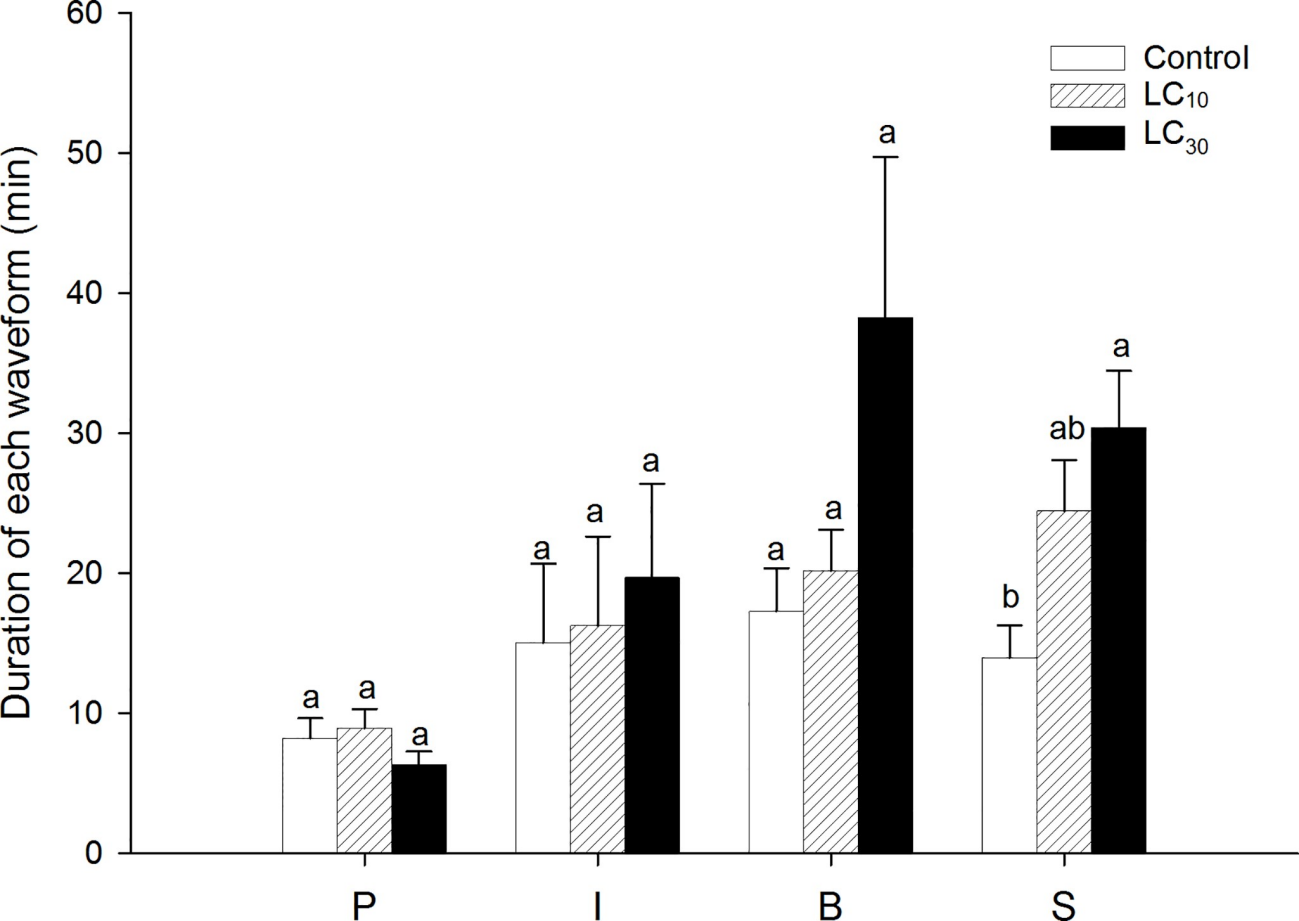
Sublethal effects of sulfoxaflor on the duration of each waveform of *Apolygus lucorum* over 6 h. P waves represent stylet probing; I waves represent insertion of stylet into cells; B waves represent cell rupturing and salivation; and S waves represent feeding on the mixture of cells. The same lowercase letters within the same parameter indicate that the treatments were not significantly different from each other based on one-way analysis of variance (ANOVA) followed by Tukey’s multiple comparisons test at *P ≤* 0.05.

Compared with the control, the number of probes significantly increased at LC_10_ (control: 6.78±1.15, LC_10_: 11.95±1.36, LC_30_: 10.74±1.18, *F* = 4.66, df = 2, 56, *P* = 0.014), as did the S (feeding cells mixture) waveform duration at LC_30_ (Fig 4, *F* = 5.63, df = 2, 56, *P* = 0.006). However, these two concentrations did not significantly affect the duration of any other waveforms from P (stylet probing), I (stylet-inserting cells), or B (cell rupturing and salivation) (Fig 4, P waveform: *F* = 0.68, df = 2, 56, *P* = 0.513; I waveform: *F* = 0.14, df = 2, 56, *P* = 0.871; B waveform: *F* = 2.56, df = 2, 56, *P* = 0.086).

## Discussion

Our results showed that the LC_10_ of sulfoxaflor caused sublethal and negative effects on the parent generation (F0) of *A*. *lucorum* by extending its nymphal development duration and reducing the oviposition period and female fecundity. This insecticide also decreased the fecundity in F0 of *Sogatella furcifera* (Hemiptera: Delphacidae) [46] and *Nilaparvata lugens* (Hemiptera: Delphacidae) [47]. Similarly, female adults of *Aphis gossypii* (Hemiptera: Aphididae) directly exposed to cycloxaprid [19] and nitenpyram [20] produced less offspring. Moreover, the LD_40_ of cycloxaprid significantly decreased the oviposition period and female fecundity of *A*. *lucorum* [16]. This phenomenon could be linked with the energy regulation in insects after insecticide exposure and more energy has been utilized by insects to cope with insecticide pressure, resulting in less energy for fecundity. However, sulfoxaflor did not affect the performance in *A*. *lucorum* [11], *Sitobion avenae* and *Rhopalosiphum padi* [48], *A*. *gossypii* [15], and *Myzus persicae* (Hemiptera: Aphididae) [38]. Thus, insects may exhibit different responses toward insecticides, which can be species-specific or affected by insecticide class and their concentrations.

Furthermore, the egg duration, oviposition period, adult longevity, and preadult survival rate significantly differ between sulfoxaflor treatment and the control in the offspring (F1) of *A*. *lucorum* (Table 2). The contrast effects of this insecticide on egg duration were similar to the finding by Tan et al. [17] who found that eggs laid by *A*. *lucorum* females exposed to LD_5_ of imidacloprid developed more slowly, but developed more quickly at LD_40_. Interestingly, this study reported that the female adult longevity of *A*. *lucorum* was significantly shorter by 7.16 days but male was longer by 6.64 days at LC_30_ of sulfoxaflor than the control. The different gender responses of *A*. *lucorum* toward cycloxaprid and imidacloprid were also confirmed by Pan et al. [16] and Tan et al. [17], respectively, and they have proposed that the bigger size and weight of females versus males may lead to such effect. However, this explanation may not be suitable here, since the stages and generation of tested insects and lethal toxicity methods were different among these studies and the topical exposure procedure adopted by them was susceptible to insect body itself. The underlying reasons need to be further explored. Additionally, both *A*. *lucorum* [11] and *Aphis glycine* (Hemiptera: Aphididae) [13, 50] have shorter oviposition period after insecticide exposure, which is consistent with our finding. The higher preadult survival rate at LC_30_ of sulfoxaflor (62%, Table 2) needs more attention and this may suggest that more adults would come out at a certain number of eggs in *A*. *lucorum*.

Life tables have been used to predict the population fitness of insects across entire life span under biotic and abiotic factors, thus avoiding the disadvantage of relying on few stages [29, 30]. In the present study, we found that the transgenerational effects of sulfoxaflor on *A*. *lucorum* depended on the tested concentrations, where the LC_30_, but not LC_10_, decreased the mean generation time (*T*) in the F1. It indicated that *A*. *lucorum* population requires less time to increase *R*_0_-fold of its size at this concentration. This was in accord with the concentration-specific effects of sulfoxaflor on the offspring of *N*. *lugens* [47]. The lower *T* was also documented in *Brevicoryne brassicae* (Hemiptera: Aphididae) exposed to LC_30_ of imidacloprid [49], *A*. *lucorum* treated with LD_15_ of sulfoxaflor [11], and *R*. *padi* with LC_25_ of sulfoxaflor [48]. In contrast, sulfoxaflor treatment increased the *T* in *A*. *gossypii* [15] and *M*. *persicae* [38]. Given that the *R*_0_, *r*, and *λ* were similar between sulfoxaflor treatment and the control (Fig 1), there will be negligible risks in inducing the population size increase in offspring of *A*. *lucorum* when their parents were exposed to this insecticide.

The disrupted feeding behaviours in response to neonicotinoid insecticides have been observed in *M*. *persicae* [14], *R*. *padi* [24, 25], *S*. *avenae* [26], *H*. *halys* [23], and *D*. *citri* [27]. Both LC_10_ and LC_40_ of cycloxaprid exerted negative effects on the phloem ingestion of *A*. *gossypii* [19]. By contrast, the count probes and number of short probes were significantly increased when *M*. *persicae* fed on plants treated with LC_30_ of imidacloprid, whereas phloem-feeding behaviour was significantly suppressed [14]. It seems that sublethal effects of neonicotinoid insecticides may increase the probe number and reduce the nutrient ingestion duration of treated insects. In this study, the number of probes and duration of feeding on cells mixture in *A*. *lucorum* were markedly lengthened by the LC_10_ and LC_30_ of sulfoxaflor, respectively. The higher number of probes may indicate that *A*. *lucorum* needs more exploration to find suitable nutritional sites, while increasing cell mixture feeding duration means that *A*. *lucorum* could get more nutrients to satisfy its own demand. Thus, the weak effects of sulfoxaflor on feeding behaviour of this insect could explain well its performance in F0 at LC_30_, while the delayed nymphal development duration and reduction in oviposition period and female fecundity at LC_10_ may be related to the energy reallocation after insecticide treatment. Meanwhile, it should be noted that only two concentrations were tested in this experiment, and further studies need to be carried out to examine whether the concentration-dependent feeding behaviour exists.

In summary, sulfoxaflor showed sublethal and concentration-specific transgenerational effects on *A*. *lucorum*, whereby LC_10_ significantly reduced nymphal development rate, oviposition period, and fecundity in F0 and LC_30_ accelerated the mean generation time in F1. This demonstrates that exposure to low concentrations of sulfoxaflor would not stimulate the resurgence of *A*. *lucorum* population. Additionally, its feeding behaviour was also weakly affected by sulfoxaflor. However, given that the controlled laboratory conditions may dramatically differ from natural conditions in the field, our findings should be carefully applied under field conditions.
